# Geospatial analysis and impact of targeted development of breast cancer care in The Gambia: a cross-sectional study

**DOI:** 10.1186/s12913-021-06963-7

**Published:** 2021-09-09

**Authors:** Ousman Sanyang, Fidel Lopez-Verdugo, Meghan Mali, Moustafa Moustafa, Jonathan Nellermoe, Justin Sorensen, Mustapha Bittaye, Ramou Njie, Yankuba Singhateh, Ngally Aboubacarr Sambou, Alison Goldsmith, Nuredin I. Mohammed, Kirstyn E. Brownson, Raymond R. Price, Edward Sutherland

**Affiliations:** 1 Center for Global Surgery, University of Utah School of Medicine, Salt Lake City, UT USA; 2 Department of Surgery, Edward Francis Small Teaching Hospital, Banjul, The Gambia; 3 School of Medicine and Allied Health Sciences, University of The Gambia, Banjul, The Gambia; 4 Department of Surgery, University of Utah School of Medicine, Salt Lake City, UT USA; 5 J. Willard Marriott Library, University of Utah, Salt Lake City, UT USA; 6 The Gambia Ministry of Health, Banjul, The Gambia; 7 Department of Obstetrics and Gynecology, University of Utah School of Medicine, Salt Lake City, UT USA; 8 Medical Research Council Unit The Gambia at the London School of Hygiene & Tropical Medicine, Fajara, The Gambia; 9 Huntsman Cancer Institute, Salt Lake City, UT USA; 10 Department of Surgery, Intermountain Medical Center, Intermountain Healthcare, Salt Lake City, UT USA; 11 Ensign College of Public Health, Eastern Region, Kpong, Ghana

**Keywords:** Breast cancer, Access to cancer care, Geospatial analysis, Global health, Global surgery, Sub-Saharan Africa

## Abstract

**Background:**

The Gambia has one of the lowest survival rates for breast cancer in Africa. Contributing factors are late presentation, delays within the healthcare system, and decreased availability of resources. We aimed to characterize the capacity and geographic location of healthcare facilities in the country and calculate the proportion of the population with access to breast cancer care.

**Methods:**

A facility-based assessment tool was administered to secondary and tertiary healthcare facilities and private medical centers and clinics in The Gambia. GPS coordinates were obtained, and proximity of service availability and population analysis were performed. Distance thresholds of 10, 20, and 45 km were chosen to determine access to screening, pathologic diagnosis, and surgical management. An additional population analysis was performed to observe the potential impact of targeted development of resources for breast cancer care.

**Results:**

All 102 secondary and tertiary healthcare facilities and private medical centers and clinics in The Gambia were included. Breast cancer screening is mainly performed through clinical breast examination and is available in 52 facilities. Seven facilities provide pathologic diagnosis and surgical management of breast cancer. The proportion of the Gambian population with access to screening, pathologic diagnosis, and surgical management is 72, 53, and 62%, respectively. A hypothetical targeted expansion of resources would increase the covered population to 95, 62, and 84%.

**Conclusions:**

Almost half of the Gambian population does not have access to pathologic diagnosis and surgical management of breast cancer within the distance threshold utilized in the study. Mapping and population analysis can identify areas for targeted development of resources to increase access to breast cancer care.

## Background

As sub-Saharan Africa (SSA) suffers from a growing burden of non-communicable diseases, cancer is being recognized as an emerging public health concern that requires attention. (1–3) Across SSA, the incidence of breast cancer is increasing, and in The Gambia incidence is increasing the most in women younger than 50 years of age, with up to 71% of cases diagnosed in this age group. (4–6) In 1986, the Gambia National Cancer Registry became one of the few nationwide population based cancer registries in SSA. (6) Current data from this registry, which is the data source for the Globocan 2020 estimates identifies breast cancer as the second most common cancer in women. (2)

A diagnosis of breast cancer in SSA often portends a poor prognosis, in part due to the 77% of black women in SSA who present with advanced disease. (7) The Gambia is estimated to have one of the lowest 5-year age-standardized relative survival rates for cancer compared to other African, Asian, and Central American countries. (8) Delayed diagnosis is one reason for advanced disease. Women in SSA wait about 3–6 months between noticing a symptom and presenting for care, and additional delays of 3–6 months occur between presentation and diagnosis. (9) Limited knowledge about breast cancer, misconceptions about the cause and available treatments, and socio-cultural factors all contribute to delayed presentation in SSA. (10) The second delay experienced, between presentation and diagnosis, reflects inefficiencies and gaps in care within the health system.

Currently in The Gambia, it is not well defined where breast cancer services are offered. The current healthcare system consists of three major tiers. (11) “Tertiary health care facilities”, which provide the highest level of care, are composed of private clinics, general hospitals and a single teaching hospital. Secondary health facilities, also called “Basic health services” include district hospitals, major health centers, and minor health centers. District hospitals are the highest functioning within this tier, but expected services are not well defined. Major health centers provide some minor surgeries, laboratory, and radiology services. Minor health centers focus on basic obstetric and child health services. “Village health services” focus on primary care and tend to minor injuries and illnesses. Understanding where breast cancer care is available within these tiers and where gaps in care exist is an important step in further developing the healthcare system to manage breast cancer. In this study, we aim to accurately delineate the locations and specific capacity of secondary and tertiary health facilities in The Gambia to screen, diagnose, and treat breast cancer and document population density surrounding each facility.

## Methods

### Study design and population

A cross-sectional, facility based survey was conducted in The Gambia from March 9, 2020 to April 9, 2020. A comprehensive list of secondary and tertiary health facilities and private medical centers and clinics was obtained from the regional health directorates. Village health services were not included in the study since these facilities are not expected to provide breast cancer care.

### Survey design

We developed a survey tool to assess the screening, diagnosis, and treatment services available for breast cancer at health facilities in The Gambia (Supplementary material). The structure was based on two existing surveys that are used to assess surgical capacity: The World Health Organization’s “Tool for Situational Analysis to Assess Emergency and Essential Surgical Care,” (WHO Tool) and Surgeons OverSeas’s (SOS) “Personnel, Infrastructure, Procedures, Equipment, and Supplies Tool” (PIPES Tool). (12, 13) The WHO Tool was the first situational analysis tool widely used to assess surgical services worldwide and has been used in over 45 low- and middle-income countries. (14) The PIPES Tool was developed as a modification to the WHO tool, but absolute numbers of personnel, hospital beds, and operating rooms were added and other questions were streamlined. (13) In their current form, neither tool was considered sufficient to assess breast cancer care capacity, as this involves multidisciplinary care that extends beyond surgery. For this reason, we developed a tool specific to breast cancer services utilizing the overall structure of the WHO and PIPES tools, including sections to inquire about human resources, infrastructure, procedures and surgeries performed, and equipment available as an outline. Local and international experts involved in breast cancer care then reviewed the tool and provided feedback. First, general information was obtained for each facility, including facility type, ownership, and whether or not the facility conducts exclusive breast clinics or sees patients with breast conditions in general clinics. Available service capacity was then assessed for each of the following topics: personnel, imaging, screening methods, diagnosis capacity, procedures and treatments performed, and follow-up.

The section on personnel assessed the number of people involved in breast cancer care in each facility. Medical doctors, physician assistants (PA), and other support staff including technicians, social workers, and nurses were included. The availability of resources and services was categorized as follows: 1) always available (resource/service available > 80% of the time over the last year), 2) not always available (resource/service available ≤80% of the time over the last year), and 3) Not available (resource/service not available at the facility).

### Survey administration

Seven research assistants (RAs), one from each of the seven health regions, were trained on the objectives of the survey, ethics, use of global positioning system (GPS), systematic and protocol-based surveying technique, and consistency in data entry. The survey tool was used in Western Region 2 (WR2) as the pilot study area. In this pilot phase, two respondents were interviewed separately per facility, each by a group of two RAs to assess consistency in survey administration. RAs were trained to use their mobile phones to obtain GPS locations of participating facilities. Location settings on their mobile phones were set to high accuracy. GIS coordinates were obtained via the native Maps app before entering the building to enhance accuracy. After completion of the pilot, RAs travelled to their assigned region for nationwide delivery.

Print and electronic copies of the tool were distributed by RAs prior to arrival at the facilities, allowing the respondents time to become familiar with the survey. Each facility identified the most suitable and knowledgeable employee to answer questions. The RAs ensured the respondent had reviewed the questionnaire and scheduled a date for the interview. GPS coordinates of the facilities were collected by the RA at the time of survey administration.

### Hospital stratification and data analysis

Descriptive data analysis was performed and results presented as frequency and proportions. Analysis was performed using Stata software (version 14.0, 2015). The National Comprehensive Cancer Network (NCCN) Framework for Resource Stratification of NCCN Guidelines, which provides recommendations for cancer care pathways based on available resources, was used as a guideline to develop a stratification system. The goal of the stratification was to objectively assess available resources for breast cancer care in The Gambia. (15) NCCN’s guidelines include three tiers: “Basic,” “Core,” and “Enhanced,” which for simplicity we labeled Level 3, 2, and 1 respectively (Table 1). (16–21) In order to better differentiate facilities that are unable to provide the full spectrum of care as described by the NCCN, we created three additional levels as follows: Level 6- facilities that provide screening and clinical diagnosis, Level 5- facilities that provide screening, clinical diagnosis, and pathologic diagnosis, and Level 4- facilities that provide screening, clinical diagnosis, pathologic diagnosis, and surgery (Table 1). Hospitals that did not fulfill the criteria for any of the mentioned levels were labelled as “other.”

**Table 1:** Criteria for stratification of healthcare facilities

Health Facility Stratification		
**LEVEL 1 (NCCN Enhanced)**	**LEVEL 2 (NCCN Core)**	**LEVEL 4**
**Screening and clinical diagnosis**	**Screening and clinical diagnosis**	**Screening and clinical diagnosis**
-Clinical breast examination -Mammography	-Clinical breast examination	-Clinical breast examination
**Pathologic confirmation & Imaging**	**Pathologic confirmation & Imaging**	**Pathologic confirmation & Imaging**
-Core needle biopsy -Skin punch biopsy -Pathological review (in house or external) -ER/PR status testing (in house or external) -HER2/neu status testing (in house or external) -Ultrasound -X Ray -Mammography -CT scan -Bone scan -Breast MRI -Genetic counseling and genetic testing	-Core needle biopsy -Pathological review (in house or external) -ER/PR status testing (in house or external) -Ultrasound -X Ray -Mammography	-Excisional biopsy, incisional biopsy, core needle biopsy, or fine needle aspiration cytology -Pathological review (in house or external) -Ultrasound -X Ray
**Surgical treatment**	**Surgical treatment**	**Surgical treatment**
-Lumpectomy -Mastectomy -Axillary dissection -Sentinel lymph node biopsy -Breast reconstruction -Oophorectomy (or medical ovarian suppression)	-Lumpectomy -Mastectomy -Axillary dissection -Sentinel lymph node biopsy -Oophorectomy (or medical ovarian suppression)	-Mastectomy (always or sometimes available)^a^ -Axillary dissection (always or sometimes available)^a^
**Non-surgical treatment**	**Non-surgical treatment**	
-Chemotherapy -Radiotherapy -Endocrine therapy -Trastuzumab -Long term surveillance/follow up -Supportive/palliative care	-Chemotherapy -Radiotherapy -Endocrine therapy -Long term surveillance/follow up -Supportive/palliative care	
	**LEVEL 3 (NCCN Basic)**	**LEVEL 5**
	**Screening and clinical diagnosis**	**Screening and clinical diagnosis**
	-Clinical breast examination	-Clinical breast examination
	**Pathologic confirmation & Imaging**	**Pathologic confirmation & Imaging**
	-Excisional biopsy or incisional biopsy -Pathological review (in house or external) -ER/PR status testing (in house or external) -Ultrasound -X Ray -Mammography	-Excisional biopsy, incisional biopsy, core needle biopsy, or fine needle aspiration cytology -Pathological review (in house or external) -Ultrasound
	**Surgical treatment**	**LEVEL 6**
	-Mastectomy -Axillary dissection -Oophorectomy (or medical ovarian suppression)	**Screening and clinical diagnosis**
		-Clinical breast examination
	**Non-surgical treatment**	
	-Endocrine therapy -Long term surveillance/follow up -Supportive/palliative care	

Table 1 Detailed list of services required to be categorized under each health facility level. A health facility must have ALL listed services to be categorized under a specific level. These services must be available > 80% of the time throughout the year unless otherwise specified. Level 6 represents a hospital with the fewest breast cancer services

^a“^Sometimes available” includes hospitals that reported offering a service, but it is only available < 80% of the time throughout the year

### Mapping, proximity analysis, reasonable travel distance, and targeted resource allocation

GIS (Geographic Information Systems) technology was employed to derive the proximity of service availability and population served within specified distances. Facility locations were geospatially visualized utilizing ArcGIS Pro software (Environmental Systems Research Institute 2020, Version 2.6), and proximity buffers extending outward in 5 km (km) increments were generated. By incorporating a 2018 LandScan population density raster obtained from the Oak Ridge National Laboratory (Oak Ridge, TN, USA), which depicts the dispersal of individuals throughout the region, a zonal statistics tool was deployed to obtain population numbers contained within each of the 5 km proximity buffers. (22) An analysis using driving distance was first attempted, but this was very limited and our GIS expert (JS) was concerned it was potentially inaccurate due to the sparse roads and barriers data available in the region. Due to this, an analysis with Euclidean (straight line) distance was performed as it has been shown to have a reasonable correlation with driving distance. (23) Patients living within a prespecified distance from the hospital were categorized as covered for that service. The results of the spatial analysis returned values for populations served within each of the specified distances while presenting a visual representation of the data.

Distance thresholds of 10 km, 20 km, and 45 km were chosen as reasonable distances for screening, pathologic diagnosis, and surgical management respectively. There are no well established thresholds for these services, so we based our distances on previously published studies that evaluated the impact of distance on utilization of health services in SSA. We also discussed reasonable travel distance with authors who have personal knowledge on transportation in The Gambia. Ten kilometers was identified as a distance predictive for utilization of screening lab services in a West African study, (24) so this was chosen as our screening distance threshold. A South African study noted that patients who had to travel greater than 20 km for care were more likely to have advanced breast cancer at diagnosis, so 20 km was chosen as our diagnosis threshold. (25) Lastly, for surgical management, a Ghanaian study identified that as travel time approached 1 hour, more than 80% of respondents would rarely or irregularly utilize available health services. (26) We chose 45 km as the surgical treatment threshold in an attempt to keep the associated travel time less than 1 h in most instances.

To observe the potential impact of a hypothetical targeted resource allocation to increase diagnosis and surgical management services, two Level 6 facilities were selected. These facilities were selected because 1) their geographic location would maximize the cumulative area of coverage with minimal overlap and 2) their staff already includes MD surgeons who could potentially be trained to provide surgical care for breast cancer. Additional maps were created and a spatial analysis was performed with these two facilities modeled as Level 4 facilities.

### Ethical approval

Ethical review was sought from the joint committee of Research and Publication Committee University of The Gambia (UTG) and Medical Research Council (MRC) Ethics committees. As this was a facility based survey with no patient involvement and no protected health information required, informed consent was given by the institution and signed by the institution’s respondent.

## Results

One hundred and two health facilities were surveyed including: the single teaching hospital in the country, seven general hospitals, four district hospitals, three major health centers, 56 minor health centers, and 31 medical centers/clinics. Fifty-nine of the facilities were owned by the state, 29 were privately owned, six were owned by faith-based organizations, four were quasi-government (i.e. community-managed facilities with partial government funding) and four responded as “other” (including three facilities managed by Non-governmental organizations and one facility funded by international institutions but primarily managed by The Gambian government). Response rate of our survey was 100%, which was accomplished because of coordination with the regional health directorates, who supported the research project and encouraged involvement from the facilities. Twenty-four reported they routinely provide breast cancer care, and the teaching hospital reported having a dedicated breast clinic.

Thirty-six health facilities, including the teaching hospital, were located in WR1, an urban region where the capital city of Banjul is located. All health regions had at least one district or general hospital, and several minor health centers. The seven health regions are further divided into 42 health districts. Five of these health districts did not have a single health facility. These health districts were located in WR2, URR, and CRR. (Table 2).

**Table 2:** Health facilities in The Gambia

Health Facilities in The Gambia				
Health Region Name	Population of Health Region (% of total population)	Number of Health Districts within Region	Number of health facilities within region	Facility Density (per 100,000 population)
**Western Region 1 (WR1)**	888,336 (40.4%)	3	36 - 1 teaching hospital - 3 general hospitals - 1 major health center - 10 minor health centers - 21 medical centers	4.05
**Western Region 2 (WR2)**	460,953 (21%)	8	14 - 1 general hospital - 1 district hospital - 1 major health center - 7 minor health centers - 4 medical centers	3.03
**Upper River Region (URR)**	269,704 (12.3%)	7	13 - 1 district hospital - 1 major health center - 9 minor health centers - 2 medical centers	4.82
**Central River Region (CRR)**	246,652 (11.2%)	11	12 - 1 general hospital - 11 minor health centers	4.86
**Lower River Region (LRR)**	86,022 (3.9%)	6	11 - 1 district hospital - 6 minor health centers - 4 medical centers	12.78
**North Bank East (NBE)**	123,623 (5.6%)	4	9 - 2 general hospitals - 7 minor health centers	7.28
**North Bank West (NBW)**	124,164 (5.6%)	3	7 - 1 district hospital - 6 minor health centers	5.63
**Total**	2,199,454 (100%)	42	102	4.63

A total number of 191 healthcare workers providing breast cancer care were reported across the 102 health facilities. Of note, 126 (66%) of these workers are in WR1. Midwives formed the largest category, with 69 employed across the country. Every region except NBE had a trained midwife providing services in breast cancer care. Twenty-two (22) medical doctor (MD) surgeons were reported in the country: 19 in WR1, two in CRR, and one in NBE. The other four regions did not have a surgeon. CRR also had three PA surgeons. There were 19 radiology personnel in the country: 11 technicians, five specialists, and three consultants located in only three regions, WR1, CRR, and NBE. The single plastic surgeon, pathology specialist, and pathology consultant in the country were all located in WR1. There were no reported medical oncology or radiation oncology personnel in the country. Sixty-seven “other” personnel included clinical residents, medical officers, house officers, and other general practitioners and nurses that were not included as a separate category in our survey.

Breast cancer screening is mainly performed via clinical breast examination (CBE), and this was always available at 52 of the 102 health facilities. Only one mammogram machine was functioning in the country and was located in a private medical center in WR1. Ultrasound was available in 11 facilities: eight in WR1, and one each in the NBE, CRR, and LRR regions. X-ray was available in 11 facilities, eight in WR1, and one each in the WR2, CRR, and NBE regions. The three computed tomography (CT) scanners and the single magnetic resonance imaging (MRI) machine in the country were all located in WR1. Positron emission tomography (PET) scans were not available.

For the pathologic diagnosis of breast cancer, seven facilities performed fine needle aspiration and excisional biopsy. Five of these facilities also performed core needle biopsy. Only one facility had in house pathology services and the other six used an external lab. All facilities offering services for pathologic diagnosis were located in WR1. Immunohistochemistry to test for estrogen receptor (ER), progesterone receptor (PR), and human epidermal growth factor receptor 2 (HER2) status was not available in The Gambia.

Seven hospitals, all located in WR1, provided surgery for the treatment of breast cancer offering both mastectomy and wide local excision. Six of the seven facilities reported performing axillary surgery, but no facilities perform sentinel lymph node biopsy. Three facilities, all in WR1, offer cyclophosphamide, methotrexate, fluorouracil (CMF) for chemotherapy, and one of these facilities also has paclitaxel and cisplatin. Breast reconstruction, radiotherapy, and endocrine therapy were not available in The Gambia. Palliative care was available at eight facilities in WR1, and one facility in WR2, CRR, LRR, NBE, and NBW.

**Fig. 1:**
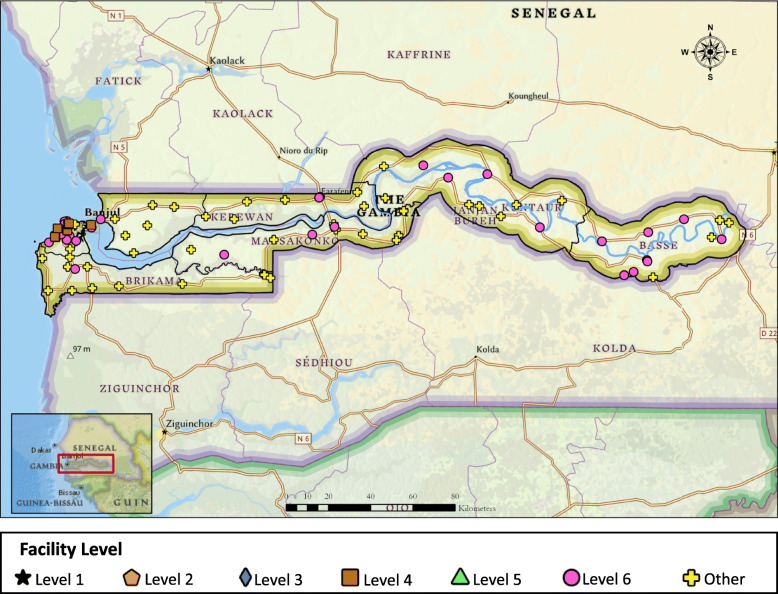
Location of healthcare facilities and level of breast cancer care provided in each facility

When the facility level stratification was applied, seven were classified as Level 4 and 38 were classified as Level 6 (Fig. 1). The majority of facilities providing breast cancer care were located in WR1 (Table 3). No facilities could provide the full spectrum of care detailed in the NCCN Framework Guidelines. The five facilities that offer the most services are all located in WR1 and are all categorized as Level 4 facilities. Three of the facilities require the addition of mammography, endocrine therapy, and testing for ER/PR status in order to provide Level 3 care. The fourth facility would require the addition of core needle biopsy, endocrine therapy, and testing for ER/PR status to provide Level 3 care.

**Table 3:** Stratification of hospitals in The Gambia

Stratification of Hospitals in The Gambia								
	WR1	WR2	URR	CRR	LRR	NBE	NBW	Total
**Level 1 (NCCN Enhanced)**	No Facilities							0
**Level 2 (NCCN Core)**	No Facilities							0
**Level 3 (NCCN Basic)**	No Facilities							0
**Level 4 (screening + path + surgery)**	7	0	0	0	0	0	0	7
**Level 5 (screening + path)**	No Facilities							0
**Level 6 (screening)**	20	1	8	4	3	1	1	38
Other*	9	13	5	8	8	8	6	57

*“Other” includes those facilities that did not fulfill criteria for any of the Levels of breast cancer care

**Fig. 2:**
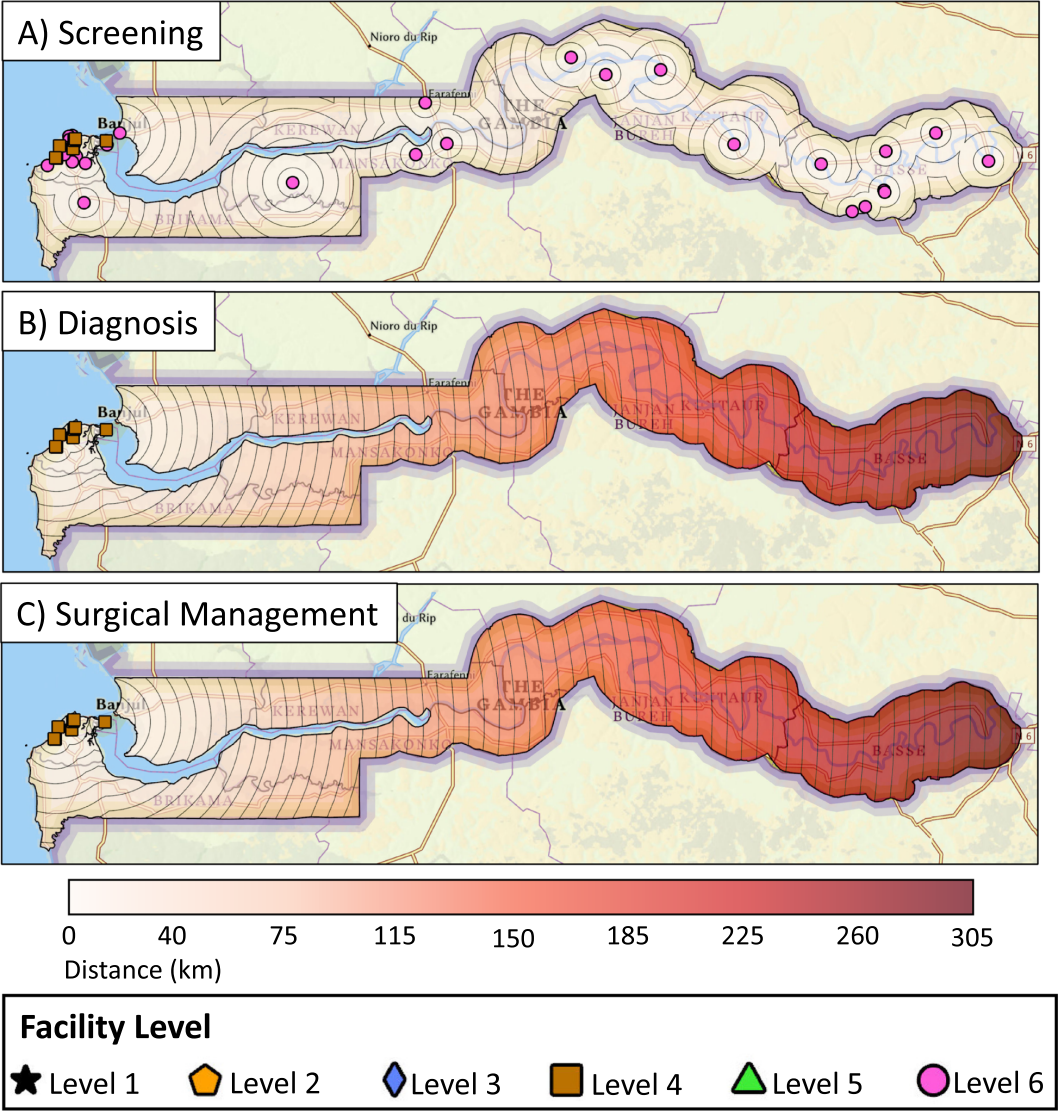
Distance from centers providing **A**) screening, **B**) diagnosis, and **C**) surgical management services for breast cancer currently

**Fig. 3:**
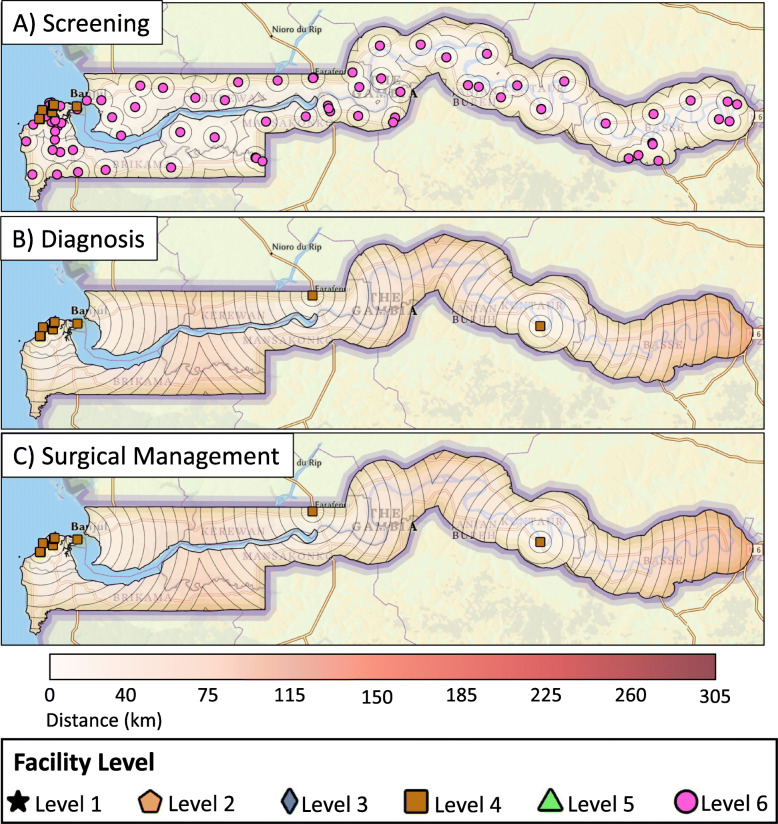
Distance from centers providing **A**) screening, **B**) diagnosis, and **C**) surgical management services for breast cancer after a hypothesized targeted resource allocation

The spatial analysis found that 72% of The Gambian population lives within 10 km of breast cancer screening with CBE, 53% lives within 20 km of pathologic diagnosis services, and 62% lives within 45 km of basic surgical care (Fig. 2). If all facilities that do not provide CBE started offering this service, 94% of the population would be within 10 km of basic screening services (Fig. 3A). If the two hospitals identified for a hypothetical targeted resource allocation as detailed above started offering diagnostic and surgical management services, the proportion of The Gambian population with geographic access to diagnosis and surgical services would increase to 62% (from 53%) and 84% (from 62%), respectively (Fig. 3B, C).

## Discussion

Improving breast cancer outcomes in a resource limited setting is challenging. Ensuring adequate geographic access to screening, early diagnosis, and treatment options is a critical step forward in improving breast cancer outcomes. In The Gambia, breast cancer care is often fragmented across different health facilities separated by wide geographical distances. With the 5 year age standardized survival for a woman with breast cancer in The Gambia being only 12%, (8) optimization of care pathways and access should be prioritized. Our study accomplished a comprehensive analysis of available services for breast cancer care and identified geographical areas most in need of expanded resources.

In this study, 38 facilities were identified that provide CBE-based screening and clinical diagnosis for symptomatic patients. Seven hospitals (all in WR1) provide pathologic diagnosis and surgical management. Limited chemotherapy is available at three facilities (all in WR1), and endocrine therapy and radiotherapy are not available in the country. We recognize that screening, pathologic diagnosis, and surgical care alone still falls short of offering the level of comprehensive breast cancer care that optimizes breast cancer outcomes in non-resource limited countries. However, given the limited resources currently available in The Gambia, the geospatial analysis focused on screening, pathologic diagnosis, and surgical care which are available and offered.

Longer distances to diagnostic and treatment facilities have been associated with delayed diagnosis and late stage at diagnosis in SSA. (27) Our results show that some patients have to travel up to 35 km for screening services, and up to 300 km for a pathologic diagnosis and surgery. This limited geographic access to care could be contributing to the current state of breast cancer in The Gambia consisting of late presentation and poor survival. (7, 8) Access to care can most simply be measured based on the presence of healthcare facilities and services in a health region, with an underlying assumption that the population of the region has access to services offered within the regional boundaries. Using this approach, the results of our survey suggest the proportion of the population with access to screening, diagnosis, and surgical treatment is 100, 40, and 40%, respectively. The 40% reflects the population in WR1 as this is the only region with diagnostic and treatment facilities in the country. Distribution of healthcare finances in The Gambia also follows this regional approach. The Directorate for Regional Health allocates finances based on reports from each region’s administrator that detail needs within the boundaries of their individual health region. Due to the small geographic size and structure of The Gambia however, expanding care services such as pathologic diagnosis and surgery in each health region may not result in the ideal distribution of resources for the country as a whole. A regional focus does not ensure access to everyone for services that have a low distance threshold, such as screening, and would result in an overlap of resources that patients are likely to travel farther for, such as surgery.

This study provides an alternative approach for resource allocation. Because Gambians are able to receive healthcare in any region, we leveraged GIS data to estimate the proportion of the population living within a pre-specified distance of healthcare facilities, rather than based on regional boundaries. Our analysis showed that current facilities provide screening, diagnosis, and surgical management to 72, 53, and 62% of the population, respectively within 10 km, 20 km, and 45 km distance thresholds. The regional approach overestimates access to screening services and underestimates access to diagnosis and surgical management. Another commonly used method to assess accessibility is the density of facilities per population. Using this metric, the density in each region ranges from 3.03 to 12.78 facilities per 100,000 population. The density observed in WR1 (4.05) is the second lowest in the country, which is inconsistent with the fact that its population has the greatest number of breast cancer services available in their region. Thus, public health policies and financial allocations based on these oversimplified estimates could result in overlooking the need for screening services in some regions and overspending to increase diagnosis and surgical management capacity in others. Our mapping could potentially guide the Ministry of Health to a more geographically equitable distribution of services while being cognizant of the constrained resources available. Additionally, this information can also be used by international partners to inform their funding.

In order to concisely define the resources available at each facility and therefore identify gaps in care, we developed a six level stratification system for the healthcare facilities. Although the NCCN “Framework” guidelines were not developed as a stratification system, their tiered structure provides an intuitive framework to classify hospitals and identify where varying resources are available. Due to the limited services currently available in The Gambia, the addition of three levels at the lower-end of the resource spectrum allowed us to highlight services available at all facilities. Recommendations regarding resource allocation can then be made using this stratification system as a guide in order to invest in the services needed to approach guideline-concordant care.

We identified two investments that could improve geographic access to screening, diagnostic, and surgical services across the country. First, in regards to screening, our survey identified that CBE was predominantly performed by midwives in facilities without medical doctors. Given that midwives were present in almost all health regions, screening and clinical diagnosis of symptomatic patients could be enhanced if midwives trained in CBE are employed at all health facilities. This would require employment of 40 CBE-trained midwives at 40 facilities that do not offer this service, as well as providing CBE training at the remaining 30 facilities that already employ midwives. These action steps would enable all facilities to provide screening services (Level 6), meaning that 95% of the population would be able to receive CBE within a 10 km radius (up from 75%). Second, in regards to pathologic diagnosis and surgical capacity, we identified two facilities that already employ surgeons where expansion of services would result in the greatest number of Gambians obtaining geographic access to care. This would require training of surgeons to perform breast biopsies and breast cancer surgery. Similar to other Level 4 facilities, pathology review of biopsied specimens could be obtained through collaboration with the teaching hospital. After this hypothetical targeted development, our population analysis suggests that a significant increase in pathologic diagnosis (53 to 62%) and surgical care (62 to 84%) could be attained.

There are a number of limitations to this study. First, barriers to accessing care consist of several dimensions and only two were assessed in this study: availability of resources and distance from care. There are many other potential barriers to accessing breast cancer care that were not evaluated, including healthcare literacy, preference for culturally aligned care, costs, road infrastructure, and travel time. (10, 28, 29) Even though these barriers were not measured, the most likely effect is that our study overestimated access, underscoring the importance of the problem. Second, Euclidean distance (straight-line) was used to estimate distance, and this approach does not account for road infrastructure. A drive time analysis would have provided a more accurate measurement of access to care as shown in previous studies. (30, 31) However due to insufficient data publicly available for this analysis, our GIS expert recommended using a Euclidean distance model as an alternative method. While Euclidean distance has demonstrated reasonable correlation with driving time, it tends to overestimate absolute population access. (23) This is an important consideration as it could lead to misclassification when using access thresholds. Third, there is no consensus on a distance threshold after which a patient can be considered as having no geographic access to care. Although we based our thresholds on the best available evidence for distance and access to healthcare in SSA, these distances are somewhat arbitrary. Finally, our survey tool has not been validated but it is based on surveys that have been widely used and previously published.

## Conclusions

In conclusion, this study provides a comprehensive assessment of available breast cancer services in The Gambia and demonstrated the use of geospatial analysis to identify gaps in care. Although comprehensive breast cancer care is not currently available in The Gambia, our stratification system was able to describe distinct levels of care currently offered. Our mapping and population analysis provides information that can be leveraged by key stakeholders to maximize return on investment in healthcare and facilitate collaborations with The Gambian Ministry of Health. Through targeted development of resources, a greater proportion of the population should have geographic access to care, which could translate to earlier diagnosis and improved outcomes.

## Data Availability

There is no additional data available. For inquiries regarding the data please contact Dr. Edward Sutherland at sutherlandmd@yahoo.com. For inquiries regarding the survey tool and possible use in another country, please contact sutherlandmd@yahoo.com or rayrprice@comcast.net.

## Abbreviations

CBE: Clinical breast examination
CMF: Cyclophosphamide, methotrexate, fluorouracil
CRR: Central River Region
CT: Computed tomography
ER: Estrogen receptor
GPS: Global positioning system
GIS: Geographic Information Systems
HER2: Human epidermal growth factor receptor 2
km: Kilometers
LRR: Lower River Region
MD: Medical doctor
MRI: Magnetic resonance imaging
NBE: North Bank East
NBW: North Bank West
NCCN: National Comprehensive Cancer Network
PA: Physician assistant
PET: Positron emission tomography
PIPES: Personnel, Infrastructure, Procedures, Equipment, and Supplies Tool
PR: Progesterone receptor
RA: Research assistant
SOS: Surgeons OverSeas
SSA: Sub-Saharan Africa
URR: Upper River Region
WHO: World Health Organization
WR1: Western Region 1
WR2: Western Region 2
